# Dietary Supplementation with n-3 Polyunsaturated Fatty Acids Delays the Phenotypic Manifestation of Krabbe Disease and Partially Restores Lipid Mediator Production in the Brain—Study in a Mouse Model of the Disease

**DOI:** 10.3390/ijms25137149

**Published:** 2024-06-28

**Authors:** Cinzia Signorini, Giovanna Pannuzzo, Adriana Carol Eleonora Graziano, Elena Moretti, Giulia Collodel, Venera Cardile

**Affiliations:** 1 Department of Molecular and Developmental Medicine, University of Siena, 53100 Siena, Italy; elena.moretti@unisi.it (E.M.); giulia.collodel@unisi.it (G.C.); 2 Department of Biomedical and Biotechnological Sciences, University of Catania, 95123 Catania, Italy; giovanna.pannuzzo74@gmail.com (G.P.); cardile@unict.it (V.C.); 3 Department of Medicine and Surgery, University of Enna “Kore”, 94100 Enna, Italy; adriana.graziano@unikore.it

**Keywords:** omega-3 PUFA, omega-3 enriched diet, fatty acid profile, brain, isoprostanes, resolvins, Krabbe disease

## Abstract

Lipid mediators from fatty acid oxidation have been shown to be associated with the severity of Krabbe disease (KD), a disorder linked to mutations in the galactosylceramidase (*GALC*) gene. This study aims to investigate the effects of n-3 polyunsaturated fatty acid (PUFA) supplementation on KD traits and fatty acid metabolism using Twitcher (Tw) animals as a natural model for KD. Wild-type (Wt), heterozygous (Ht), and affected Tw animals were treated orally with 36 mg n-3 PUFAs/kg body weight/day from 10 to 35 days of life. The end product of PUFA peroxidation (8-isoprostane), the lipid mediator involved in the resolution of inflammatory exudates (resolvin D1), and the total amount of n-3 PUFAs were analyzed in the brains of mice. In Tw mice, supplementation with n-3 PUFAs delayed the manifestation of disease symptoms (*p* < 0.0001), and in the bran, decreased 8-isoprostane amounts (*p* < 0.0001), increased resolvin D1 levels (*p* < 0.005) and increased quantity of total n-3 PUFAs (*p* < 0.05). Furthermore, total brain n-3 PUFA levels were associated with disease severity (r = −0.562, *p* = 0.0001), resolvin D1 (r = 0.712, *p* < 0.0001), and 8-isoprostane brain levels (r = −0.690, *p* < 0.0001). For the first time in a natural model of KD, brain levels of n-3 PUFAs are shown to determine disease severity and to be involved in the peroxidation of brain PUFAs as well as in the production of pro-resolving lipid mediators. It is also shown that dietary supplementation with n-3 PUFAs leads to a slowing of the phenotypic presentation of the disease and restoration of lipid mediator production.

## 1. Introduction

In cell membranes, the lipid profile is mainly understood as the composition of fatty acids, e.g., polyunsaturated fatty acids (PUFAs), long- or short-chain PUFAs, omega-3 (n-3) or omega-6 (n-6) PUFAs, has been studied in neurological diseases [1,2]. In neurological disorders in particular, but not only [3,4], the relevance of n-3 PUFAs and n-6 PUFAs to the pathological condition has been investigated, and the effects of dietary interventions aimed at altering the ratio of n-6 PUFAs to n-3 PUFAs have been assessed [1,2,5].

The brain is enriched in lipids. Docosahexaenoic acid (DHA, 22:6n-3), one of the most abundant PUFAs in the brain, together with arachidonic acid (AA, 20:4n-6) [6], has been evaluated as a beneficial molecule for reducing cell death, for recovering from damage and improving cognitive function [7,8,9,10,11,12,13,14], and for assuring the physiological brain development [15,16,17], although DHA effects are still debated and need to be explored in depth [18,19]. In addition, eicosapentaenoic acid (EPA, 20:5n-3), an n-3 PUFA, is thought to regulate brain function [14], improve memory and attention [12,13], and maintain brain volume [13]. Thus, EPA’s relevance in maintaining healthy brain function is emphasized. Moreover, n-3 PUFAs have been shown to reduce recurrent epilepsy seizures [20,21,22], even if the blood increase, on a genetic basis, of PUFAs was found to be associated with an increased risk of epilepsy [23]. EPA and DHA are the long-chain PUFA derivatives of alpha-linolenic acid (ALA, 18:3n-3). Frequently, the more abundant PUFAs in the diet are linoleic acid (LA, 18:2n-6) and ALA. LA and ALA are not synthesized in animals and are regarded as essential fatty acids [4,24]. Overall, the ratio of n-3 to n-6 PUFAs is considered a critical factor for the regulation of neurophysiological and cognitive functions [6].

In Krabbe disease (KD), because of mutations in the galactosylceramidase (*GALC*) gene, deposition of the toxic lipid D-glucosyl-beta1-1′-sphingosine (GluSph) in the brain occurs [25]. The Twitcher mouse (Tw) is an enzymatically authentic model of human KD. It is deficient in GALC enzyme activity, shares many of the neuropathological and biochemical features of human KD, is the most commonly used animal model to study KD pathogenesis and is used for experimental therapies [25,26,27]. Heterozygous (Ht) mice carry one copy of a mutation that causes KD-like disease. As in human KD, the disease is rapidly progressive in the Tw mouse model, and interventional approaches have been tested to prevent significant damage to myelinating cells [28,29]. In Tw mouse, neurological symptoms appear between 15 and 20 days after birth and include tremors, progressive paralysis of the hind limbs, and loss of coordination [30,31]. Interestingly, Ht mice have been studied and compared to Tw animals [26] to better understand the increased risk of developing a number of different diseases in Ht carriers. In the murine Tw model, brain levels of oxygenated PUFA metabolites (i.e., isoprostanes, IsoPs) and pro-resolving lipid mediators, in particular resolvin (Rv)D1 derived from DHA, have been found to be relevant for the severity of the disease [32,33]. Interestingly, a lipidomic approach to studying the nervous system was performed in homozygous Tw mice, and it was found that lipid classes differed significantly from those of wild-type mice [34]. Moreover, a specific fatty acid profile was found in Tw mice compared to wild–type (Wt) mice [35], and a mechanism has been described in which psychosine toxicity in KD is associated with membrane lipid raft disruption and membrane microsimulation [36].

In this study, we used the Tw mouse as a KD model to investigate whether dietary n-3 PUFAs can modulate brain n-3 PUFA content, brain PUFA oxidation, and KD severity. The study was performed in Wt control mice, Ht carriers of the *GALC* mutation, as unaffected mice, and affected Tw mice fed with or without dietary supplementation of n-3 PUFAs.

## 2. Results

### 2.1. Disease Severity Scoring

**Figure 1:**
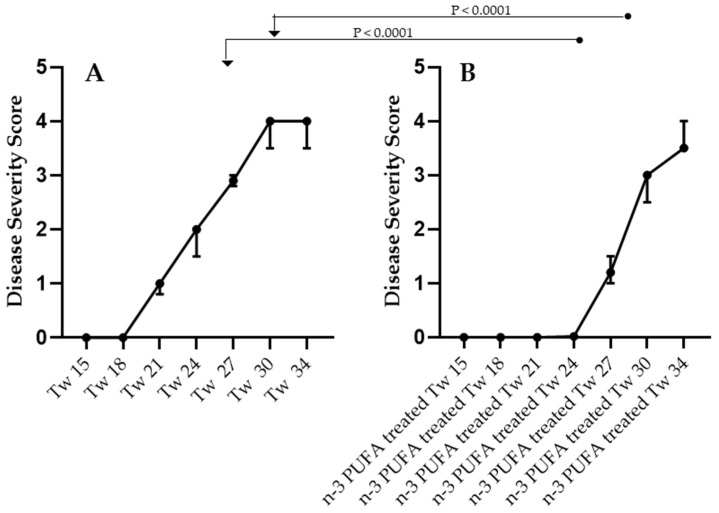
Disease severity score was recorded from day 15 to day 34 in both Tw mice. Panel (**A**) Tw mice without n-3 PUFA supplementation; panel (**B**), Tw animals supplemented with n-3 PUFAs. Data are expressed as medians (points); bars are interquartile ranges. The differences between the groups were compared using the nonparametric Kruskal–Wallis test (*p* < 0.0001, 14 groups, n = 210) followed by the nonparametric Mann–Whitney test (significant *p* values are displayed in the figure). The displayed statistical comparisons are referred to as n-3 PUFA treated Tw 27 vs. Tw 27 and n-3 PUFA treated Tw 30 vs. Tw 30. The number of animals was n = 15 for each group. Legend: Tw, twitcher; the associated number (15, 18, 21, 24, 27, 30, 34) indicates the day of score recording. In the name of the group, “treated” stands for “supplemented”.

In the study, each animal was assigned a score between 0.0 and 4.0 according to the phenotypic characteristics using the scoring criteria described in Materials and Methods (Severity score of disease). A higher score meant a higher severity of the disease. In the Tw mice, the disease severity scores assigned to each animal on days 8, 15, 21, 24, 30, and 34 were found to be significantly different. (ANOVA test, *p* < 0.0001, n = 210). In particular, it was found that the time of onset of phenotypic disease was delayed in Tw mice under n-3 PUFA treatment. On days 27 and 30, the severity of the disease was significantly lower (*p* < 0.0001) in the n-3 PUFA supplemented Tw than in the unsupplemented (no n-3 PUFA supplementation) mice. Although supplemented (n-3 PUFA supplemented Tw) and unsupplemented Tw mice showed no significant difference in disease severity on the day before sacrifice (day 34), the time of delay in disease symptoms as a result of n-3 PUFA supplementation was evident (Figure 1).

### 2.2. RvD1 in KD Brain

**Figure 2:**
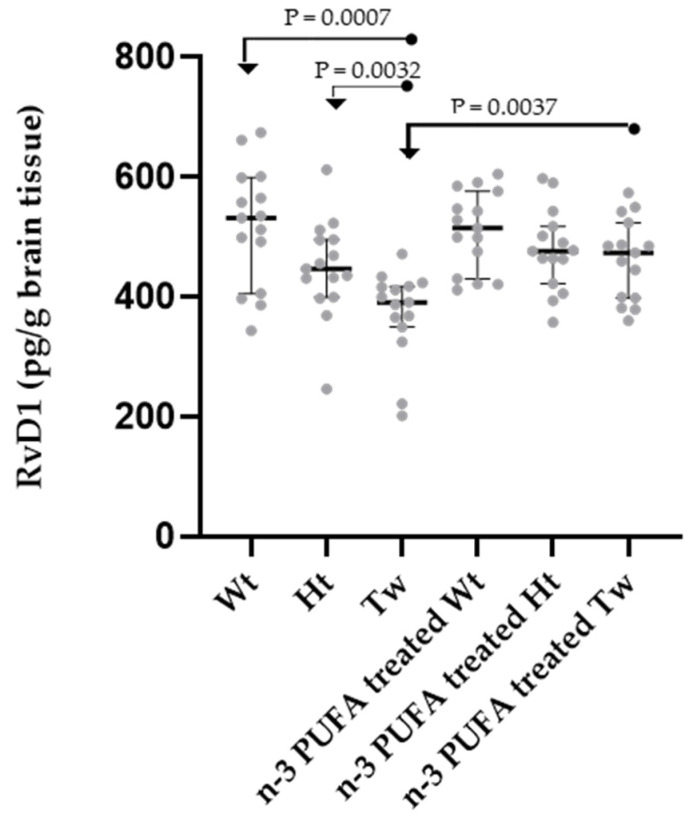
Dot plots of RvD1 brain levels in n-3 PUFA supplemented or non-supplemented wild-type (Wt), heterozygous (Ht), and twitcher (Tw) mice. Differences between groups were compared using the nonparametric Kruskal–Wallis test (*p* = 0.0001, six groups, n = 90), followed by the nonparametric Mann–Whitney test (significant *p* values are displayed in the figure). For each experimental group, the dots represent individual data, horizontal lines represent the medians, and the error bars represent the 95% interquartile range. A description of the statistical results is reported in the text. The number of animals was n = 15 for each group. Legend: Wt, wild-type; Hz, heterozygotes; Tw, twitcher. In the name of the group, “treated” stands for “supplemented”.

RvD1 values detected in brain tissues in all the experimental groups (Wt, Ht, Tw, n-3 PUFA Supplemented Wt, n-3 PUFA Supplemented Ht, n-3 PUFA Supplemented Tw) were compared by multiple comparison analysis test (ANOVA test) and were found significantly different (ANOVA test, *p* < 0.0001, n = 90). The evaluation of RvD1 levels in brain tissue shows a significant RvD1 reduction in Tw mice compared to Wt and Ht animals under basal conditions (i.e., without supplementation with n-3 PUFAs) (Figure 2). Notably, the amount of RvD1 in the brain was shown to be decreased with the increase of phenotypical manifestations in KD mice.

In mice supplemented with n-3 PUFAs, Tw and Ht show an increase in RvD1 levels. Remarkably, RvD1 brain levels are significantly increased in Tw mice treated with n-3 PUFA compared to Tw animals without n-3 PUFA supplementation, whereas RvD1 amounts in Tw animals supplemented with n-3 PUFAs are not significantly different from those of Wt animals supplemented with n-3 PUFAs and non-supplemented Wt animals. The RvD1 levels in the brains of Ht and Tw animals supplemented with n-3 PUFAs also did not differ. Thus, supplementation with DHA and EPA achieves a relevant improvement in the production of the lipid mediator (RvD1), which is mainly involved in the resolution of the inflammatory process (Figure 2).

### 2.3. F_2_-IsoP in KD Brain

**Figure 3:**
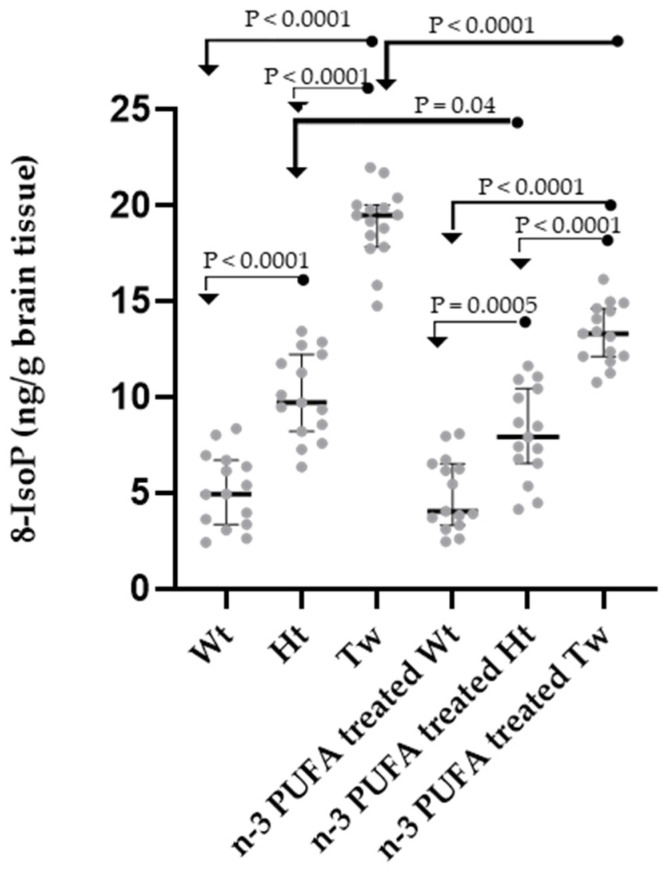
Dot plots of 8-IsoP brain levels in n-3 PUFA supplemented or non-supplemented wild-type (Wt), heterozygous (Ht), and twitcher (Tw) mice. Differences between groups were compared using the nonparametric Kruskal–Wallis test (*p* < 0.0001, six groups, n = 90), followed by the nonparametric Mann–Whitney test (significant *p* values are displayed in the figure). As an additional and not displayed significant comparison, n-3 PUFA Supplemented Tw were different from Wt (*p* < 0.0001). For each experimental group, the dots represent individual data, horizontal lines represent the medians, and the error bars represent the 95% interquartile range. A description of the statistical results is reported in the text. The number of animals was n = 15 for each group. Legend: Wt, wild-type; Hz, heterozygotes; Tw, twitcher. In the name of the group, “treated” stands for “supplemented”.

Eight-IsoP, a representative isomer of F_2_-IsoP compounds as described above, was analyzed in the brain homogenate of all animals (Figure 3). The amounts of 8-IsoP measured in each experimental group were compared and found to be statistically different (ANOVA test, *p* < 0.0001, n = 90). Although significant differences are maintained between Wt, Ht, and Tw within each group (with and without supplementation), some significant differences are highlighted due to the n-3 PUFA supplementation performed. As the most important result, a significant decrease in brain 8-isoP levels was observed in the Tw-treated group compared to the untreated Tw mice (difference between medians = 6.15, *p* < 0.0001). In addition, brain 8-IsoP levels were also significantly lower in the PUFA-treated Ht animals compared to non-supplemented Ht mice (difference between medians = 1.80, *p* = 0.04).

The 8-IsoP levels in Tw and Ht mice supplemented with n-3 PUFAs do not decrease with respect to the levels found in Wt animals (supplemented or not) but are significantly reduced compared to the corresponding non-supplemented Tw and Ht mice. Therefore, following n-3 PUFA supplementation, a partial and significant reduction in lipid damage occurs, even if the amounts observed in the Wt control mice are not restored.

### 2.4. n-3 and n-6 PUFA Contents in Brain Tissue

**Figure 4:**
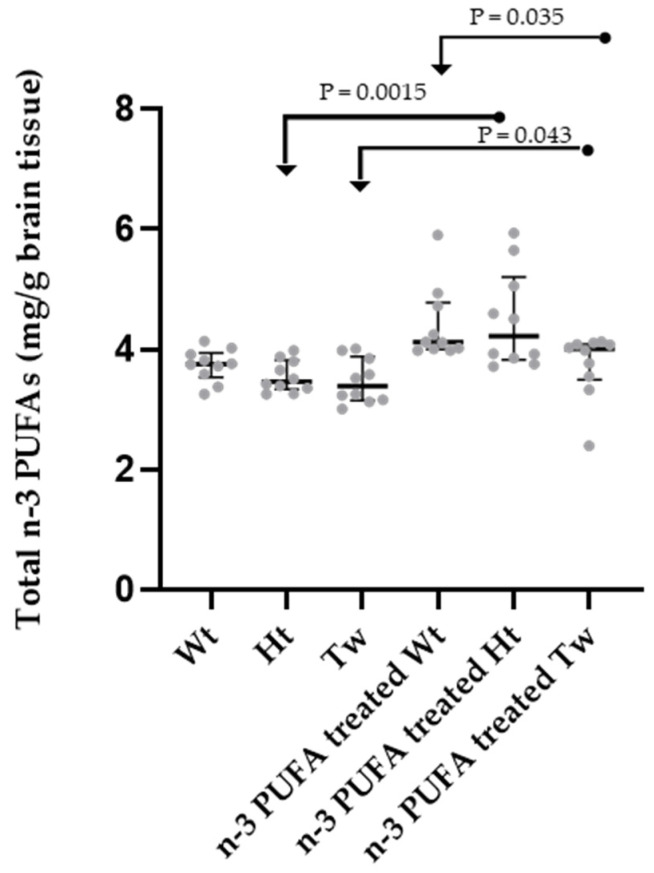
Dot plots of brain amounts of n-3 PUFAs in wild-type (Wt), heterozygous (Ht), and twitcher (Tw) mice supplemented and non-supplemented with n-3 PUFAs. Differences between groups were compared using the nonparametric Kruskal–Wallis test (*p* < 0.0001, six groups, n = 60), followed by the nonparametric Mann–Whitney test (significant *p* values are shown). Additional and not shown significant comparisons: Wt differed from n-3 PUFA supplemented Wt (*p* = 0.0011); n-3 Supplemented Ht vs. Wt (*p* = 0.0185). For each experimental group, the dots represent individual data, horizontal lines represent the medians, and the error bars represent the 95% interquartile range. A description of the statistical results is reported in the text. The number of animals was n = 10 for each group. Legend: Wt, wild-type; Hz, heterozygotes; Tw, twitcher. In the name of the group, “treated” stands for “supplemented”.

When evaluating the total amount of n-3 PUFAs in the brain tissue, statistical differences were found between the test groups (ANOVA test, *p* < 0.0001, n = 60). The applied n-3 PUFA supplementation induced an increase in n-3 PUFA brain levels compared to the corresponding non-supplementation state. Such an increase was more pronounced in Tw and Ht than in Wt (Tw = Ht > Wt) (Figure 4).

In brain tissue, total n-6 PUFA levels were measured. No difference was detected when Wt, Ht, and Tw animals were compared, and no effect of n-3 PUFA treatment was found. In the brain, total n-6 PUFA levels ranged from 2.88 to 2.95 (median values, mg/g brain tissue) in the non-supplemented groups and from 2.93 to 3.31 (median values, mg/g brain tissue) in the n-3 PUFA treated animals. In addition, no significant difference was observed for the n-3/n-6 PUFA ratio both before and after n-3 PUFA supplementation, with the value of the ratio (medians) ranging from 1.13 to 1.8 in the non-supplemented animals and from 1.22 to 1.48 in the treated mice.

### 2.5. Correlation of Disease Severity, RvD1, and 8-IsoP to n-3 PUFAs

**Figure 5:**
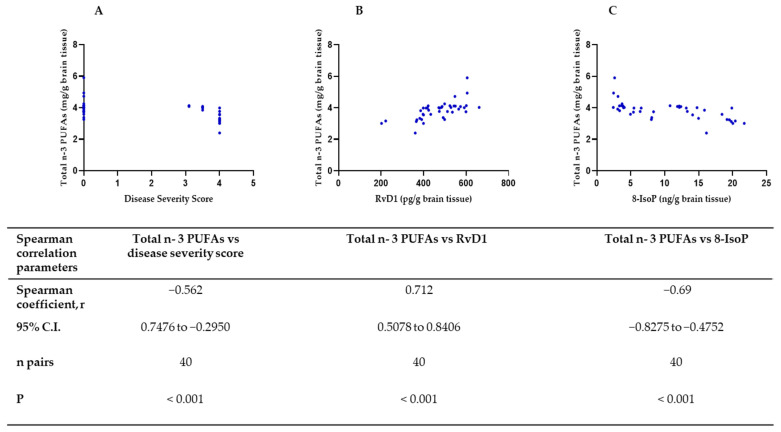
Relationship between disease severity, RvD1, and 8-IsoP, and the total amount of n-3 PUFAs in brain tissue. (**A**) Scatter plot for n-3 PUFA content in brain tissue and disease severity. Several animals had the same severity. Scores were assigned as described in Methods. (**B**) Scatter plot for brain RvD1 content and total n-3 PUFAs. (**C**) Scatter plot for 8-IsoP and total n-3 PUFAs. Both supplemented and non-supplemented n-3 PUFA cases were included. The relationship between each pair of variables was tested using the Spearman correlation test. The Spearman coefficient (r), the lower and upper limits of the 95% confidence intervals (C.I.), the number of data pairs (n pairs), and the *p*-values are shown.

In all animals studied, the total amount of n-3 PUFAs in the brain correlated significantly with the estimated disease severity, RvD1, and 8-isoP levels in the brain. Figure 5 shows the data for all animals with and without n-3 PUFA supplementation.

Remarkably, the level of n-3 PUFA in brain tissue was found to be significantly correlated with brain levels of 8-IsoP, disease severity, and RvD1 brain levels both before and after n-3 PUFA supplementation (Table 1).

## 3. Discussion

Oxidative stress and inflammation are known to contribute to the onset and progression of chronic degenerative diseases. PUFAs, which modulate both antioxidant signaling pathways and inflammatory processes, are widely implicated in the pathophysiology of chronic diseases. In addition, n-3 PUFAs, particularly EPA and DHA, have been associated with a lower incidence of chronic inflammatory diseases [4,37,38]. Here, these issues were investigated in KD, where free radical-triggered fatty acid oxidation and impairment of resolution of inflammation are considered relevant factors in KD brain pathology [33].

Supplementation with DHA and EPA slows the phenotypic manifestation of the disease and largely normalizes the biochemical processes involved in the enzymatic and free radical-triggered oxidation of PUFAs. In fact, 8-IsoP levels, although significantly reduced compared to the non-supplemented Tw, in supplemented Tw do not reach the levels found in the Wt control animals, whereas the RvD1 values in the supplemented Tw normalize to the values measured in the Wt control mice, both supplemented and non-supplemented animals. Considering this result, Tw mice probably benefit from supplementation with n-3 PUFAs because these stimulate the resolution of inflammation. Furthermore, it is necessary to consider that 8-IsoP derives from the oxidation of arachidonic acid, while RvD1 derives from the enzymatic metabolism of DHA. Therefore, multiple aspects of lipid oxidative metabolism are presumably implicated in the symptoms of KD and might be differently modulated by n-3 PUFA supplementation.

Increases in pro-resolving mediators and beneficial changes in circulating pro-resolving mediators were found as results of a clinical trial after marine n-3 fatty acid supplementation [39]. Moreover, nutritional supplementation with n-3 PUFAs and antioxidants was also associated with good maintenance of cognitive status in patients with mild cognitive impairment [40,41]. The relevance of n-3 PUFA supplementation in the management of chronic inflammatory pathologies was documented in several different clinical conditions, even in conditions where the intake of fatty acids must be limited, such as, for example, in hepatic steatosis [42]. This reinforces the concept that the supplementation with n-3 PUFAs is to be considered as a supply of fatty acids able to support the resolution of inflammation, determining a resolvin up-regulation. Thus, also in the investigated Tw model, where a specific fatty acid profile was detected in the brain and serum of Tw mice [35], the n-3 PUFA supplementation is here demonstrated to be useful in modulating the severity of the disease, the oxidative lipid damage and the production of a mediator for the resolution of inflammation. It is suggested that therapeutic approaches that promote the resolution of inflammation rather than block inflammation (anti-inflammatories) may be warranted [43,44], and in this sense, there are numerous efforts to target production or receptors of the specialized pro-resolving mediators, among which RvD1 is included. RvD1 has also already been shown to resolve neuroinflammation after brain damage, and in hemicerebellectomy, a reduced ability to produce RvD1 has been reported [45]. Moreover, RvD1 treatment was found to ameliorate neuroinflammation and improve neurological function neuroinflammation in different conditions [46,47]. The relevance of specialized pro-resolving mediators from fatty acid oxidation in neurological and psychiatric disorders has been deeply investigated, beneficial effects have been reported, and therapeutic strategies have been suggested [48]. In KD, neuroinflammation is observed as in most leukodystrophies and coincides with white matter pathology [49]. Thus, it is not surprising that there is an interest in the role of dietary PUFAs in ameliorating such diseases through the production of useful lipid mediators. Pharmacological combined protocols, including n-3 PUFAs and pro-resolving mediators, could be able to interact at multiple levels in the cross-talk of the pathophysiological mechanisms. The ability of lipids to cross the blood-brain barrier, as well as the availability of a suitable vehicle to deliver drugs to treat neurological disorders [50], can support the increase in total n-3 PUFA levels shown in the brain tissue of the experimental model investigated. The ability of n-3 PUFAs to cross the blood-brain barrier has been investigated, and it was shown that in aging, the modification of the metabolism of peripheral plasma n-3 PUFAs modifies the amount of n-3 PUFAs available for uptake by the brain [51].

To underline the possibility of using supplementation with n-3 PUFAs in neurological diseases, appear to be relevant the studies concerning the effects of n-3 PUFA supplementation in a further genetic neurological disease. In an early stage of Rett syndrome (OMIM#312750), oral supplementation with fish oil (containing DHA and EPA) of patients led to a significant reduction in clinical severity along with a significant decrease in oxidative damage of fatty acids and the reduction of fatty acid anomalies present in the erythrocyte membranes of subjects affected by Rett syndrome [1,2].

## 4. Materials and Methods

### 4.1. Animals

As previously described, the Tw mouse is a current model of human KD. Pairs of mice carrying the *GALC* mutation (W339X) were crossed repeatedly to obtain a progeny of TW mice. The diseased mice were generated according to the probability of 25% (i.e., 1 in 4 pups) for each offspring; therefore, the affected Tw animals here investigated belonged to different litters. The animals were housed in plastic cages in our facility at the Department of Biomedical and Biotechnological Sciences (University of Catania), following the provisions of community and national legislation, and were fed a standard granulated feed supplemented with n-3 PUFAs, as described below, and tap water ad libitum.

The mice were sacrificed on the 35th day of life, close to the death time of the affected Tw animals.

The Institutional Animal Care and Use Committee approved all experimental procedures involving animals in accordance with institutional guidelines for animal care and use (Project no. 364; authorization no. 61/2022-PR).

### 4.2. Severity Score of Disease

In Krabbe-affected mice (Tw mice), clinical symptoms develop at the onset of the active myelination period, and if untreated, the lifespan is approximately 35 days.

Starting from the 15th day of the life of the animals, the body weight was monitored every 3–4 days, and the evaluation of coordination tests, closure of the hind limbs, gait, and onset and severity of tremor was carried out [32]. As previously described [32], the severity of the disease was calculated and assigned for each parameter assessed (coordination tests, hindlimb closure, gait, and tremor) using a scoring system consisting of a scale from 0 to 4. In this scoring system, a score of 0 was assigned for the absence of the KD phenotype, while a score of 4 was assigned for a severe expression of the disease signs assessed. All the individual scores were combined to attribute a single final score to each animal. The described evaluation procedure was able to detect both differences in disease severity between strains and differences over time within the same strain.

### 4.3. Diet

To study the effects of a diet supplemented with n-3 PUFAs, mice fed a standard diet and, starting from the 10th day, aliquots of a para-pharmaceutical Omega 3 fish oil, 90% minimum content of n-3 PUFAs (Fagron Omega 3 Forte 60prl, Italian Ministry Registration Code: 923513838, Fagron Italia Srl, Quarto Inferiore, Bologna, Italy), containing the ethyl esters of DHA (240 mg/g) and EPA (510 mg/g) were administered orally to Ht and Tw animals to treat with 36 mg of n-3 PUFAs/kg body weight/day, an amount known to have beneficial effects [52,53,54]. A group of Wt mice was equally supplemented with n-3 PUFAs (36 mg/kg body weight/day).

Furthermore, additional Wt, Ht, and Tw animals were on a standard diet as control groups.

In detail, animals were grouped as follows:

1th group: healthy mice on standard diet (n = 15) (Wt in Figure 2, Figure 3 and Figure 4);

2nd group: unaffected heterozygous mice on standard diet (n = 15) (Ht in Figure 2, Figure 3 and Figure 4);

3rd group: affected twitcher mice on standard diet (n = 15) (Tw in Figure 1, Figure 2, Figure 3 and Figure 4);

4th group: healthy mice, on standard diet and n-3 PUFA supplementation (n = 15) (n-3 PUFA Supplemented Wt in Figure 2, Figure 3 and Figure 4);

5th group: unaffected heterozygous mice, on standard diet and n-3 PUFA supplementation (n = 15) (n-3 PUFA Supplemented Ht in Figure 2, Figure 3 and Figure 4);

6th group: affected twitcher mice, on a standard diet and −3 PUFA supplementation (n = 15) (n-3 PUFA Supplemented Tw in Figure 1, Figure 2, Figure 3 and Figure 4).

### 4.4. Collection of Animal Brain Samples

At the time of the sacrifice, on the 35th day (postnatal day, terminal course of the disease), mice were sacrificed with CO_2_, and then, the brains were removed and immediately stored at −80 °C until further experiments. At the time of analysis, the whole brain was homogenized in phosphate-buffered saline (PBS), pH 7.4 (10% *w*/*v*).

### 4.5. Fatty Acid Assessment in Brain Tissue

Fatty acids were determined in whole brain tissue, previously homogenized in phosphate-buffered saline (PBS), pH 7.4 (10 % *w*/*v*). As previously reported for erythrocytes or spermatozoa [2,55], phospholipids were first extracted and then converted into fatty acid methyl esters (FAMEs) by an alkali-catalyzed transesterification reaction in a methanol solution of 0.5 M KOH [56,57,58]. The FAMEs obtained were then analyzed by a gas chromatograph (Agilent 7890B, Agilent Technologies, Milan, Italy) using a capillary column (60 m × 0.25 mm × 0.20 μm, Rtx-2330, Restek, Milan, Italy) and a flame ionization detector. Details of gas chromatography conditions: injector temperature, 250 °C; temperature started from 50 °C, held for 1 min, followed by an increase of 25 °C/min up to 175 °C, held for 3 min, followed by a second increase of 4 °C/min up to 230 °C, and held for 30 min.; constant pressure mode (25.9 psi); hydrogen as carrier gas (flow, 1.89 mL/min); injector temperature 250 °C, volume injection 1 μL, splitess mode. The identification of methyl esters was performed by comparing them with authentic molecules. All the determinations were performed at the Lipidomics Laboratory, Lipinutragen Srl, CNR Area della Ricerca di Bologna, Italy.

### 4.6. Resolvin (Rv) D1 Immunoassay

In brain tissue (10% *w*/*v*, whole brain homogenate in PBS, pH 7.4), RvD1 levels were immunodetected (Cat No. MBS2600566, MyBio-Source, San Diego, CA, USA). Briefly, a sandwich technique, defined as double because more than two possible antigen epitopes can be identified by both the pre-coated capture antibody and the detection antibody simultaneously, was applied. A biotin-labeled antibody and a horseradish peroxidase-avidin conjugate were used. Sample optical density for absorbance measurement was evaluated at 450 nm. A reference curve (authentic RvD1 amounts between 2000 pg/mL and 31.2 pg/mL) was applied for sample RvD1 level determination, and the results were expressed as pg/g brain tissue.

### 4.7. F_2_-Isoprostane (F_2_-IsoP) Determination in Mouse Brain

F_2_-IsoPs are shown as reliable biomarkers of PUFA oxidation, mainly by non-enzymatic mechanisms, also in neurological diseases [59,60,61].

F_2_-IsoPs, commonly detected as 8-isoprostane (8-IsoP) is one F_2_-IsoP abundantly produced in vivo by the oxidation of arachidonic acid [62,63], were previously reported to be relevant to KD [32]. Here, 8-isoprostane was measured in whole mouse brain tissue in its total amount (including 8-isoprostane released in free form and 8-isoprostane esterified in lipids). Thus, sample hydrolysis was performed by adding 1 mM KOH solution (500 μL) to an aliquot (1 mL) of brain homogenate and incubating at 45 °C for 45 min. Subsequently, each sample was acidified, adding HCl (1 mM, 500 μL). The subsequent solid phase extraction was carried out by applying onto a C_18_ cartridge (500 mg Sorbent per Cartridge, 55–105 µm Particle Size, 6cc, Waters, Milford, MA, USA) that was preconditioned with methanol (5 mL) and water (5 mL). After the deposition of the sample, the cartridge was washed with 10 mL water (pH 3); finally, 8-IsoP was eluted with methanol (5 mL). Eight isoprostanes were immunologically detected by a competitive immunoassay (Item No. 516351, Cayman Chemical, Ann Arbor, MI, U.S.A.). A reference curve was applied to determine 8-IsoP levels in each sample, and the results were expressed as ng/g brain tissue.

### 4.8. Statistical Analysis

The D’Agostino-Pearson normality test was applied to assess the normality of data. Multiple comparisons were carried out by one-way analysis of variance (ANOVA), with the Kruskal–Wallis test followed by the nonparametric Mann–Whitney test, on account of non-normal data distribution. The association between variables was tested using the Spearman rank correlation at 95% confidence intervals (95% C.I.). A two-tailed *p* ≤ 0.05 was considered to indicate statistical significance. The Graph-Pad Prism 8.4.2 statistical software package was used for the data analysis.

## 5. Conclusions

**Figure 6:**
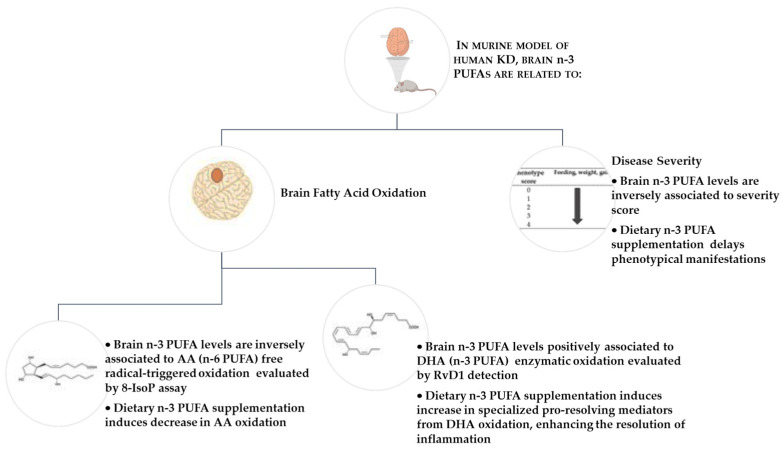
In a murine model of KD, total n-3 PUFAs in the brain are related to oxidative fatty acid metabolism in the brain and to the disease severity. Brain amounts of n-3 PUFAs are negatively related to both the disease severity score and 8-IsoP levels, whereas they are positively related to RvD1 brain levels. In symptomatic twitcher mice, dietary supplementation with DHA and EPA (both n-3 PUFAs) delays disease phenotypical manifestation, decreases lipid peroxidation in the brain, and increases the production of RvD1, which is a specialized mediator for inflammatory resolution. Legend: 8-IsoP, 8-isoprostane; AA, arachidonic acid; DHA, docosahexaenoic acid; RvD1 resolvin D1; n-3 PUFA, omega-3 polyunsaturated fatty acids; KD, Krabbe disease. Created with BioRender.com and PowerPoint 2013 (Microsoft Office Professional Plus 2013, Redmond, WA, USA).

The total amount of n-3 PUFAs in the brain tissue of the KD mouse model is related to disease severity, PUFA peroxidation, and the production of pro-resolving lipid mediators (Figure 6). Dietary supplementation with n-3 PUFA slows the phenotypic manifestation of the disease and rebalances fatty acid oxidative processes by decreasing free radical-induced oxidation and increasing enzymatic oxidation, leading to the production of bioactive lipid mediators that play an important role in regulating inflammatory processes.

## Figures and Tables

**Table 1:** Data for Spearman correlation between n-3 PUFA content in the brain and disease severity, RvD1 or 8-IsoP, before and after n-3 PUFA supplantation.

Spearman Correlation	Before n-3 PUFA Supplementation	After n-3 PUFAs Supplementation
n-3 PUFAs vs. disease severity	Spearman coefficient r = –0.63 95% C.I.: −0.8414 to −0.2424, n pairs = 20 *p* = 0.003	Spearman coefficient r = –0.66 95% C.I.: −0.8557 to −0.2896 n pairs = 20 *p* = 0.002
n-3 PUFAs vs. RvD1	Spearman coefficient r = 0.70, 95% C.I.: 0.3486 to 0.8723 n pairs = 20 *p* < 0.001	Spearman coefficient r = 0.70, 95% C.I.: 0.3486 to 0.8723 n pairs = 20 *p* < 0.001
n-3 PUFAs vs. 8-IsoP	Spearman coefficient r = –0.68, 95% C.I.: −0.8671 to −0.3296 n pairs = 20 *p* < 0.001	Spearman coefficient r = –0.84, 95% C.I.: −0.9363 to −0.6223 n pairs = 20 *p* < 0.001

The Spearman coefficient (r), the lower and upper limits of the 95% confidence intervals (C.I.), the number of data pairs (n pairs), and the *p*-values are shown.

## Data Availability

All authors had full access to all the data in the study and took responsibility for the integrity of the data and the accuracy of the data analysis. All data used to support the findings of this study are included within the article, and all data are available.
